# Quantification of the heat wave effect on mortality in nine French cities during summer 2006

**DOI:** 10.1371/currents.RRN1307

**Published:** 2012-03-12

**Authors:** Mathilde Pascal, Alain Le Tertre, Abdessattar Saoudi

**Affiliations:** Institut de Veille Sanitaire, Département Santé Environnement, Saint Maurice, France

## Abstract

Background: July 2006 was the first major heat wave in France after the creation of a heat prevention plan. Understanding its impacts on health will help improving the efficiency of this plan. We assessed the mortality impact of the heat wave, and investigated the influence of the heat prevention plan.

Methods: The study focused on nine French cities. A Poisson regression model was used to analyze the correlation between temperature, air quality and mortality. An additional spline of time was introduced to capture an additional heat wave effect. Heat-action days defined by the prevention plan were introduced as a dummy variable.

Results: 411 extra deaths were observed in the nine cities during the 2006 heat wave. Unlike the 2003 heat wave, no additional heat wave effect was observed in 2006. The maximum daily relative risk of mortality varied from 1.45 in Strasbourg (IC 95% [1.01-2.08]) to 1.04 in Lille (IC 95% [0.92-1.18]). The impact on mortality of the implementation of heat-action days was non-significant and highly variable depending on the cities, with a combined excess of relative risk of -3.3% (IC 95% [-10.3%; 4.4%]).

Conclusions: Although no specific heat wave effect was observed, warm temperatures and air pollution were still responsible for a significant excess mortality in France. The absence of a specific heat wave effect may be partly explained by the prevention plan. It may also indicate that higher temperatures are required to observe a mortality outburst.

## Background 

In France, past heat waves have been characterized by an excess mortality and morbidity among elderly people, workers, patients suffering from chronic diseases and infants [1] [2]. The warmest heat wave was experienced in 2003, when the excess mortality was estimated to be around 14 800 between the 1^st^ and 20^th^ August 2003 (+60%) [1]. This event showed no evidence of short-term mortality displacement [1] [3]. To prevent similar events, a national prevention plan was developed in 2004 by the French Ministry of Health.   

The impact of this national heat prevention plan on the reduction of the risks, and on the excess mortality and morbidity during heat waves is still to be determined. A key limitation to this evaluation is the lack of events since 2004. The main one occurred in July 2006. Minimum and maximum temperatures were below those observed during the August 2003 heat wave, but July 2006 was the warmest month of July in France since 1950. Using a nation-wide model, it was estimated that if the conditions have been those prevailing before 2003, 6 452 excess deaths should have been recorded during the July 2006 heat wave, while about 2 100 excess deaths were observed [4]. This discrepancy may be interpreted as a decrease in the population's vulnerability to heat, together with increased awareness of the risks related to extreme temperatures, preventive measures and the set-up of the heat prevention plan.  

However, this nation-wide model does not allow taking into account spatial heterogeneity, while in 2003 the impact was found to be extremely variable between cities, with the highest burden paid in Dijon, Le Mans, Lyon and especially Paris (+142% excess mortality for summer 2003) [5]. The heterogeneity is likely to be larger in 2006 than in 2003, due to the geographic spread of the heat wave, and to the fact that the prevention plan was implemented differently in the different cities. An analysis of the impact of the 2006 heat wave at the city level is thus required to gain a better understanding of the mortality response during a heat wave, and of the role of the prevention plan. A key question is to understand if the sustained heat during several days generated an additional burden to the one caused by day-by-day temperatures. Indeed, an analysis of the 2003 heat wave showed that the mortality response during the heat wave was exceptional compared to the temperature-mortality relationship usually observed in France. Models based on temperature only did not fit correctly the mortality peak during the 2003 heat wave. An additional term was then introduced in these models to fit the specific mortality response during the heat wave [6]. It is worth investigating if such effect was still observed in 2006, when the prevention plan was available.  

In this paper, we assessed the mortality impact of temperature and ozone during summer 2006 in nine French cities, investigating for an additional effect, and for the influence of the warnings and the implementation of the heat prevention plan.  

## Material and methods  

### Study area 

This study was set in nine French cities; Bordeaux, Le Havre, Lille, Lyon, Marseille, Paris, Rouen, Strasbourg and Toulouse. 

### Mortality data 

For each city, all causes daily mortality data (International Classification of Diseases, 10^th^ Revision codes A00–R99), were obtained from the National Institute of Statistics and Economic Studies (Insee) for the period 2000-2006.  

### Temperature and air pollution data 

For the same period, daily minimum and maximum temperatures were obtained from the national meteorological office, Météo-France. Ozone concentrations (8 hours maximum values) were obtained from the local air monitoring networks.    

### Information on the implementation of the heat prevention plan 

During summer, heat wave periods are anticipated using temperature forecasts from the meteorological services Météo-France. An alert is issued when two temperature indicators (minimum temperature averaged over 3 days and maximum temperature averaged over 3 days) have a high probability of being above minimum and maximum thresholds. These thresholds vary geographically; a meteorological station, usually located in the main city, has been chosen per department, and is used to give a warning for the whole department [7]. This warning can lead to the implementation of preventive actions during some days, labelled as heat-action days. Preventive actions during heat-action days include social services calling or visiting vulnerable people, increase staff in hospitals and nursing homes, or dissemination of advices through radio and television spots. Yet, apart from the legally binding actions, no data are available about actions that are effectively implemented at the local level by the different stakeholders (authorities, health professionals, NGOs, employers…). Therefore, information on the dates of warnings and on the implementation of a heat-action days was collected for each city to create a binary variable (0 no action, 1 actions), without further detailed on the implemented actions. 

### Statistical analysis 

We applied a method previously used for the analysis of the 2003 impacts in the same cities, detailed elsewhere [6]. We used a generalised additive model (GAM) with a Poisson distribution that allows for over-dispersion to model the variation of the daily mortality data. We controlled for possible confounders, including long-term trend, season, days of the week, bank holidays, minimum temperature on the current day, maximum temperature on the previous day, and O_3_ - 8 hour mean of the current and the previous days (0–1 day lag), following the APHEA-2 (Air Pollution and Health: a European Approach) methodology [8]. The degree of smoothing of the spline function for season and long-term trend was chosen to minimize autocorrelation in the residuals. The temperatures were modelled using 3 degrees of freedom. To explore a possible additional effect, we added a penalized cubic regression spline of time which covers a period of 47 days (27/06-11/08). This period was chosen to well capture the increase of mortality during the heat wave and to allow the analysis of a potential short term mortality displacement. The degree of smoothing of this spline function was again chosen to minimize autocorrelation in the residuals. The daily relative risk of the specific effect of the heat wave was computed as the number of expected deaths estimated by the heat wave spline divided by the number of expected deaths estimated in the absence of a heat wave. The number of deaths in the absence of the heat wave was predicted using references values for temperature and ozone, computed as the mean of the values observed between 2000 and 2005, excluding 2003.     The overall number of excess deaths was the difference between the number of expected deaths estimated by the heat wave and the number of expected deaths in the absence of the heat wave.   

We also investigated the potential effect of heat action plan on mortality by adding the heat-action days as a 0/1 variable in the models. A meta-analysis was conducted to evaluate the impact of this variable.  

Data were analysed using the MGCV package [9] in the R software. 

## Results 

 The nine cities have a total population of about 11 millions, ranging from 6 million in Paris to 254 585 in Le Havre. The mean daily mortality varied from 5.5 in Le Havre to 94.4 in Paris. The proportion of deaths occurring among people over 65 years ranged from 73% in Lille to 81% in Marseille. Mean minimum temperature varied from 6.6°C in Strasbourg to 10.8°C in Marseille, and mean maximum temperature from 13.9°C in Le Havre to 20.1°C in Marseille. Demographic and climatic characteristics of the cities are presented in Table 1.  

      **Table 1 - Demographics characteristics, daily mortality, summer (June-August) temperatures and 8h-ozone concentrations**

**Table:** 

**City**	**Population**	**Daily mortality**		**Tmin (°C)**		**Tmax (°C)**		**Ozone (ppb)**	
		**Mean**	**Max**	**Mean**	**Max**	**Mean**	**Max**	**Mean**	**Max**
**Bordeaux**	584 164	12.2	29	9.3	22.9	18.2	38.3	68.2	160.5
**Le Havre**	254 585	5.5	15	9.2	21.8	13.9	33.3	64.0	162.2
**Lille**	1 091 156	21.0	41	7.4	22.6	14.5	33.9	57.8	182.1
**Lyon**	782 828	17.1	41	8.4	24.6	17.0	38.3	64.5	184.8
**Marseille**	856 165	21.9	45	10.9	24.7	20.1	37.6	79.1	191.8
**Paris**	6 164 418	94.4	171	9.0	21.9	15.8	35.6	56.6	193.1
**Rouen**	434 924	9.3	23	6.8	19.7	14.3	33.2	61.3	186.3
**Strasbourg**	451 133	7.2	20	6.6	21.3	15.4	36.0	60.1	185.9
**Toulouse**	690 162	11.2	33	9.2	23.1	18.2	38.1	72.7	161.6

The 2006 heat wave was less intense than the 2003-heat wave, and had a large geographical heterogeneity. Between the 11^th^ and the 27^th^ July 2006, minimum temperatures above 19°C and maximum temperatures above 34°C were observed in all cities (Table 2). Maximum temperature reached 38.3°C in Toulouse, 37.3°C in Bordeaux and 37°C in Marseille. However, these levels were well below the temperature observed in 2003, then reaching 39 to 40.5°C. Figure 1 compares the distribution of the mean temperature anomalies in July and August i.e. the differences between the daily mean temperatures and the usual values, defined as the means of the daily temperatures observed between 2000 and 2006 (excluding August 2003 and July 2006). While during the August 2003 heat waves, temperatures were outliers in all cities, during the July 2006 heat waves, temperatures were exceptional (i.e. higher than the 95^th^ percentile of the temperature distribution) only in Bordeaux, Le Havre, Lille and Rouen. 

In 2006, the highest ozone concentration was observed in Lille, with levels that were well below those observed in 2003 (Table 2).  

**Table 2 – Warning thresholds, minimum and maximum temperatures averaged over 3 days and 8h-ozone concentrations between the 11** ^**th**^ **and 27** ^**th**^ **July 2006**

**Table:** 

**City**	**Warning thresholds (°C)**		**Tmin (3 days)**		**Tmax (3 days)**		**Ozone (ppb)**	
	**Tmin (3days) **	**Tmax ** **(3days) **	**Mean**	**Max**	**Mean**	**Max**	**Mean**	**Max**
**Bordeaux**	21	35	20	23	34	37	118.7	142.5
**Le Havre**	19	33	18	21	26	32	120.7	163.3
**Lille**	18	33	17	19	30	34	126.7	192.1
**Lyon**	20	34	21	23	34	37	150.7	181.2
**Marseille**	24	35	23	24	34	36	146.2	164.9
**Paris**	21	31	20	22	32	35	132.2	189.6
**Rouen**	19	33	16	19	29	33	120.7	163.3
**Strasbourg**	19	34	18	20	32	35	148.3	184.6
**Toulouse**	21	36	21	22	34	37	88.8	127.9

**Figure:**
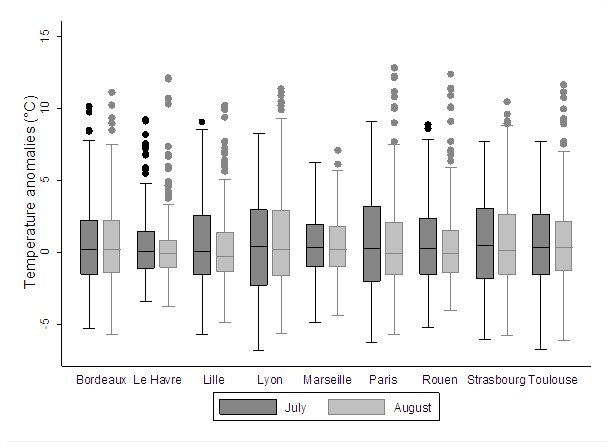
**** **Figure 1 – Mean Temperature anomalies in July and August per cities between 2000 and 2006 (in July, dots correspond to the extreme of temperatures during the 2006 heat wave, and in August to extreme of temperatures during the 2003 heat wave)** 

In the national prevention plan, a heat wave is characterised by a sustained period of minimum and maximum temperature above specific thresholds.  Based on observed temperatures, these thresholds were reached in only 4 of the 9 cities investigated. However, because forecasted temperatures may be over-estimated, and because decision-makers can decide to maintain an alert even when the temperatures have fallen below the thresholds, warning periods do not correspond to the strictly observed heat wave periods (Table 3). Heat-action days were decided for more than ten days in most cities. They were consistent with the observed temperatures in Bordeaux, Lyon, Paris and Strasbourg. On the opposite, in Marseille and Toulouse, warnings were issued, but the temperature thresholds were not reached.  

 The excess relative risk associated to the implementation of heat-action days was non-significant and highly variable between cities.  The implementation of heat-action days was associated to a combined loss of relative risk of mortality of -3.3% (IC 95% [-10.3% - +4.4%]) (Table 3). 

**Table 3 – Heat wave and alert period between the 27/06-11/08 2006 per city, % increase in mortality during the heat-action days**

**Table:** 

**City**	**Heat wave period** **(observed temperature > thresholds)**	**Heat-action days**	**% increase in mortality during heat-action days **
**Bordeaux**	14 - 21 July	16- 27 July	13.1 [-12.5 : 46.3]
**Le Havre**	no	no	-
**Lille**	no	no	-
**Lyon**	18 - 28 July	01-05 July, 18-29 July	-11.9 [-27.2 : 6.6]
**Marseille**	no	30 June - 05 July, 07 July - 2 August	-8.9 [-29.4 : 17.6]
**Paris**	19-21 July, 24-27 July	01-05 July, 17-28 July	-4.4 [-13.7 : 5.9]
**Rouen**	no	no	-
**Strasbourg**	24-27 July	19-29 July	1.6 [-27.8 : 43.0]
**Toulouse**	no	16-18 July, 24-28 July	13.8 [-15.3 : 53.1]

For each of the nine cities, Figure 2 presents the variation of the relative risk of mortality of the heat-wave effect between the 27/06/2006 and the 11/08/2006. Compared to the results obtained in 2003, no specific effect of the heat wave was observed in 2006. The maximum daily relative risk varied from 1.45 in Strasbourg ([1.01-2.08]) to 1.04 in Lille [0.92-1.18] (Table 4). In all cities, the variations of the mortality observed in the cities during summer 2006 were explained by the usual daily variations of the ozone and temperature.    

 

**Figure:**
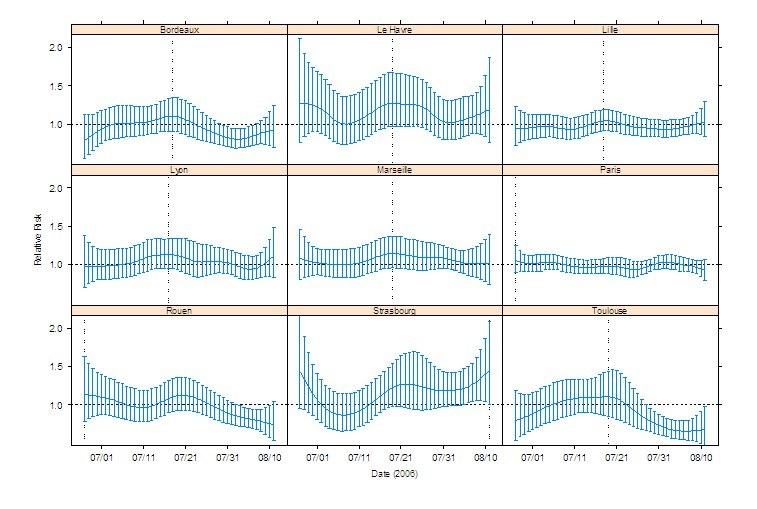
**Figure 2 - Relative risk associated to the heat wave and 95% confidence interval per city between the 27/06/2006 and the 11/08/2006 (dash lines indicate the days with the maximum RR).**   

**Table 4 – Maximum relative risk per city between the 27/06-11/08 2006, and date of the maximum risk**

**Table:** 

**City**	**RR**_**max**_	**IC RR**_**max**_ ** (95%)**	**Date**
**Bordeaux**	1.1	[0.92; 1.33]	2006-07-18
**Le Havre**	1.28	[0.97; 1.49]	2006-07-19
**Lille**	1.04	[0.92 ; 1.18]	2006-07-18
**Lyon**	1.13	[0.97; 1.31]	2006-07-17
**Marseille**	1.14	[0.98 ; 1.33]	2006-07-19
**Paris**	1.05	[0.9 ; 1.23]	2006-06-27
**Rouen**	1.13	[0.78 ; 1.29]	2006-06-27
**Strasbourg**	1.45	[1.01 ; 2.08]	2006-08-11
**Toulouse**	1.1	[0.88; 1.39]	2006-07-19

The high temperatures and ozone concentrations resulted in 411 excess deaths between the 27/06 and the 11/08 2006 compared to the 2000-2005 average (2003 excluded) (Table 5). Le Havre and Strasbourg present the highest excess mortality, respectively +15% and +10%. A small harvesting effect was observed mainly in Paris, Lyon and Lille.  

**Table 5 – Excess mortality between the 27/06-11/08 2006 per city**

**Table:** 

**City**	**Excess deaths associated to the temperature, ozone and heat wave effect**	**Excess deaths associated to the heat wave **	**Harvesting**	**Net excess mortality**	**Net excess mortality rate per 100 000 inhabitants **
**Bordeaux**	52	44	-7	37	6.3
**Le Havre**	35	44	0	44	14.5
**Lille**	26	28	-42	-39	- 3.4
**Lyon**	8	9	-38	-30	- 4.7
**Marseille**	82	49	-6	43	4.3
**Paris**	85	28	-67	-40	- 0.6
**Rouen**	11	27	-41	-14	- 8.5
**Strasbourg**	62	36	-11	25	8.2
**Toulouse**	50	32	-47	-15	- 5.4
**Total**	411	272	259	12	0.3

## Discussion 

The 2006 summer was warmer than usual and high temperatures were observed in all cities. However, the criteria for defining a heat wave according to the heat prevention plan were reached only in 4 out of the 9 cities studied. In all cities, we did not observe a specific heat wave effect during the 2006 heat wave, and variations of the mortality were explained by the usual daily variations of the ozone and temperatures. 

 The high levels of temperature and ozone were responsible for 411 deaths in the nine cities between the 27/06 and the 11/08 2006. Half of the deaths (207) occurred in the four cities were the temperatures exceeds the warning thresholds (Bordeaux, Lyon, Paris and Strasbourg). These results are consistent with the analysis at the national level, describing a lower than expected, but still significant impact of the 2006 heat wave (2 100 excess deaths during the heat wave period) [4]. 

 This new study also provides further insight into the geographical heterogeneity of the heat wave and of its impacts.  In comparison, in 2003, the criteria defining a heat wave were reached in 8 of the 9 same cities. 3 096 extra deaths were recorded in summer 2003, and maximum daily relative risks of mortality during the heat wave ranged from 1.16 in Le Havre to 5.00 in Paris [6]. Le Havre is the only city where the maximum risk of mortality was higher in 2006 than in 2003 (resp 1.28 and 1.16), and where the mortality burden was higher in 2006 (resp 14.5 per 100 000 inhabitants vs - 8.4 per 100 000 inhabitants in July). It is worth underlying that the temperatures were not the warmest in Le Havre compared to other cities, but that they were very unusual compared to the temperatures usually observed in that city in July.   

In Bordeaux, Lyon, Paris and Strasbourg were heat waves were identified both in 2003 and 2006, differences in the heat wave intensity or duration might be an explanation of the absence of a heat-wave effect in 2006. The hypothesis of a heat wave effect observed only when intensity and duration exceeds a certain value is consistent with the results obtained in the EuroHeat project, where a larger impact was found for long heat waves, or heat waves lasting several days and characterized by extreme temperatures  [10]. Consistently, in the US it was found that most excess risk observed during heat waves was comparable to the independent effects of individual days with the same temperatures.  A small specific heat wave effect was observed only when the heat waves lasted more than 4 days [11]. Therefore, we can make the hypothesis that for moderate heat waves, the usual temperature-mortality relationship is observed, while an additional effect is observed during extreme heat waves. We used the terms moderate and extremes in reference to the usual temperatures variations observed in specific cities. Considering the example of Le Havre, the relative intensity of the heat wave seems to be more important than its absolute intensity.  

 Differences may also be explained by the prevention measures implemented during the 2006 heat waves. In France, the heat prevention plan is tailored to respond to those very exceptional heat waves. Our results confirm that a significant mortality burden was still observed during the 2006 heat wave, even in cities where the observed temperatures remained below the heat warning thresholds. Therefore, efforts to promote short and long-term prevention must be maintained, with an enhance communication of heat-related risks and appropriate behaviours all through summer. We already have indications that the heat prevention plan has changed the awareness of the heat-related risks in the general population. A questionnaire send to 1240 adults aged over 15 showed that 74% of the people had heard, read or seen heat wave prevention materials during the summer. 63% of the people had taken protective measures during the 2006 heat wave, and 73% had taken measure to protect their elderly relatives and friends, including regular visits (39%) and regular phone call (29%) [12]. 

Considering all the factors influencing the mortality response during a heat wave, the lower than expected mortality burden observed in 2006 does not allow concluding on the efficiency of the heat prevention plan. However, it is worth underlying the low mortality response observed in those cities where the temperatures exceeded the heat wave thresholds and where heat-action days have been implemented. We found that the implementation of heat-action days was non-significant and highly variable depending on the cities, with a combined excess of relative risk of -3.3% (IC 95% [-10.3%; 4.4%]). The variable heat-action days cover a variety of situations; days when temperatures were really above the thresholds, days when forecasts have been over-estimated and observed temperatures were lower than expected, days when the temperatures were below the thresholds and stakeholders had yet decided to activate the plan. Actions implemented during these days also varied between cities. It is thus not surprising that a large heterogeneity and large confidence intervals are associated with this variable. In addition, as heat action days were activated during most of the heat wave periods in Lyon, Bordeaux and Strasbourg, this variable was highly correlated with the heat wave variable, which limits the capacity to interpret the results. Since 2006, a procedure has been introduced to reduce the impact of forecasting uncertainty on the warning decision, and we have a better knowledge of the action implemented at the local level. Data of better quality should be available to investigate the role of the prevention plan in the analysis of future heat waves. 

## Competing interest declaration

The authors have declared that no competing interests exist.      

## Funding information

None

## References

[ref-3530677870] Fouillet A, Rey G, Laurent F, Pavillon G, Bellec S, Guihenneuc-Jouyaux C, Clavel J, Jougla E, Hémon D. Excess mortality related to the August 2003 heat wave in France. Int Arch Occup Environ Health. 2006 Oct;80(1):16-24. Epub 2006 Mar 8. 16523319 10.1007/s00420-006-0089-4 PMC1950160

[ref-2815774959] Rey G, Jougla E, Fouillet A, Pavillon G, Bessemoulin P, Frayssinet P, Clavel J, Hémon D. The impact of major heat waves on all-cause and cause-specific mortality in France from 1971 to 2003. Int Arch Occup Environ Health. 2007 Jul;80(7):615-26. Epub 2007 Feb 14. PubMed Central PMCID: PMC2291483. 17468879 10.1007/s00420-007-0173-4 PMC2291483

[ref-1191115861] Filleul L, Cassadou S, Médina S, Fabres P, Lefranc A, Eilstein D, Le Tertre A, Pascal L, Chardon B, Blanchard M, Declercq C, Jusot JF, Prouvost H, Ledrans M. The relation between temperature, ozone, and mortality in nine French cities during the heat wave of 2003. Environ Health Perspect. 2006 Sep;114(9):1344-7. 16966086 10.1289/ehp.8328 PMC1570046

[ref-857300186] Fouillet A, Rey G, Wagner V, Laaidi K, Empereur-Bissonnet P, Le Tertre A, Frayssinet P, Bessemoulin P, Laurent F, De Crouy-Chanel P, Jougla E, Hémon D. Has the impact of heat waves on mortality changed in France since the European heat wave of summer 2003? A study of the 2006 heat wave. Int J Epidemiol. 2008 Apr;37(2):309-17. Epub 2008 Jan 13. PubMed Central PMCID: PMC2652641. 18194962 10.1093/ije/dym253 PMC2652641

[ref-2163740764] Vandentorren S, Suzan F, Medina S, Pascal M, Maulpoix A, Cohen JC, Ledrans M. Mortality in 13 French cities during the August 2003 heat wave. Am J Public Health. 2004 Sep;94(9):1518-20. PubMed Central PMCID: PMC1448485. 15333306 10.2105/ajph.94.9.1518 PMC1448485

[ref-1726721064] Le Tertre A, Lefranc A, Eilstein D, Declercq C, Medina S, Blanchard M, Chardon B, Fabre P, Filleul L, Jusot JF, Pascal L, Prouvost H, Cassadou S, Ledrans M. Impact of the 2003 heatwave on all-cause mortality in 9 French cities. Epidemiology. 2006 Jan;17(1):75-9. 16357598 10.1097/01.ede.0000187650.36636.1f

[ref-3979596377] Pascal M, Laaidi K, Ledrans M, Baffert E, Caserio-Schönemann C, Le Tertre A, Manach J, Medina S, Rudant J, Empereur-Bissonnet P. France's heat health watch warning system. Int J Biometeorol. 2006 Jan;50(3):144-53. Epub 2005 Nov 23. 16328399 10.1007/s00484-005-0003-x

[ref-4244347560] Touloumi G, Samoli E, Pipikou M, Le Tertre A, Atkinson R, Katsouyanni K; APHEA-2 Project Group. Seasonal confounding in air pollution and health time-series studies: effect on air pollution effect estimates. Stat Med. 2006 Dec 30;25(24):4164-78. 16991105 10.1002/sim.2681

[ref-2877912105] Wood SN. 2006. Generalized Additive Models: An Introduction with R.

[ref-3665683147] D'Ippoliti D, Michelozzi P, Marino C, de'Donato F, Menne B, Katsouyanni K, Kirchmayer U, Analitis A, Medina-Ramón M, Paldy A, Atkinson R, Kovats S, Bisanti L, Schneider A, Lefranc A, Iñiguez C, Perucci CA. The impact of heat waves on mortality in 9 European cities: results from the EuroHEAT project. Environ Health. 2010 Jul 16;9:37. PubMed Central PMCID: PMC2914717. 20637065 10.1186/1476-069X-9-37 PMC2914717

[ref-391334742] Gasparrini A, Armstrong B. The impact of heat waves on mortality. Epidemiology. 2011 Jan;22(1):68-73. 21150355 10.1097/EDE.0b013e3181fdcd99 PMC3324776

[ref-46859090] Léon C, Girard D, Arwidson P, Guilbert P. 2007. Comportements préventifs des Français et impact des campagnes de prévention durant la canicule 2006. Evolutions 7:1-6.

